# The Bottom Line: A Multidisciplinary Approach Is Key to Treating Perianal Fistulizing Crohn’s Disease

**DOI:** 10.1093/jcag/gwy063

**Published:** 2018-11-14

**Authors:** Geoffrey C Nguyen

**Affiliations:** Mount Sinai Hospital Centre for Inflammatory Bowel Disease, University of Toronto, Toronto, Ontario, Canada

This is in reference to the article by Steinhart AH, Panaccione R, Targownik et al., ‘Clinical Practice Guideline for the Medical Management of Perianal Fistulizing Crohn’s Disease: The Toronto Consensus.’

The management of Crohn’s disease (CD) can be highly variable depending on disease phenotype and presentation. Perianal fistulizing CD occurs in up to one-quarter of CD patients and remains one of the more challenging complications of this chronic disease to treat (1). The clinical presentation of perianal disease can range from simple fistula to life-threatening pelvic sepsis, and a spectrum of current therapies spans the gamut from antibiotics to fecal diversion. Importantly, the presence of perianal disease portends poor outcomes in CD and can substantially compromise quality of life. The Canadian Association of Gastroenterology has developed guidelines to foster a systematic approach to managing this fistulizing CD (2). The American Gastroenterological Association (AGA) and the World Gastroenterology Organization (WGO) have also published guidelines on this topic within the last several years (3–5). However, the CAG guidelines are the only ones that fully adapt the Grading of Recommendations Assessment, Development and Evaluation (GRADE) system to formulate a strength of recommendation. The strength of recommendation (strong versus weak/conditional) has important implications for physicians as they discuss treatment options with their patients. A strong recommendation should be carried out universally and has medicolegal reverberations. In contrast, a conditional recommendation allows significant discretionary judgment. This distinction is particularly important since the quality of evidence for clinical studies in perianal disease has been rated as low or very low across the board.

The CAG consensus panel provided a strong endorsement of magnetic resonance imaging or endoscopic ultrasound to characterize the anatomy of perianal fistula. Regardless of the classification system (e.g., Parks, AGA Technical Review), knowing the location and type of fistula may guide both medical and surgical management. For this reason, positioning it as a diagnostic intervention that should be performed in most patients is reasonable. The most significant challenge in implementation will be access to these diagnostic modalities. In Canada, endoscopic ultrasound remains largely confined to academic centers. Pelvic MRI is more widely available, but the wait times vary regionally and can be substantially longer than other imaging modalities (e.g., ultrasound, CT). From a practical standpoint, clinicians will not wait for the results of imaging before starting antibiotic therapy and may request other modalities, such as pelvic CT, to rule out pelvic abscess if warranted.

In addition to antibiotics, anti-TNF therapy is a cornerstone of medical therapy. Its use was the other strong recommendation offered by the CAG consensus group. Though the quality of evidence supporting the effectiveness of anti-TNF therapy was downgraded due to imprecision and inconsistency, it represents the best line of evidence available out of all the current medical therapies for perianal fistula. Importantly, anti-TNF agents can maintain fistula closure once achieved. Though this is a strong recommendation, it should be noted that some patients with simple perianal fistula may be sufficiently treated with a course of antibiotics and may not require anti-TNF therapy. The panel additionally suggested the use of anti-TNF therapy in combination with either thiopurines or methotrexate, mostly extrapolating data from luminal CD. This combination therapy may serve to improve anti-TNF drug levels, which can be advantageous for the treatment of perianal fistulizing CD because higher trough levels may be required than for luminal disease.

The CAG guidelines, however, made no mention of thiopurine or methotrexate monotherapy for the treatment of perianal fistulizing CD, which starkly contrasts the strong recommendation for anti-TNF therapy. This positioning of anti-TNF therapy in these guidelines distinguishes it from algorithms for treating luminal CD. Provincial drug coverage programs, particularly in Ontario, frequently require a trial of immunomodulator therapy before approving anti-TNF agents for fistulizing CD. Referencing these guidelines and their strong recommendation for anti-TNF therapy will hopefully facilitate access to anti-TNF therapy earlier in the course of treatment.

One of the key messages from these guidelines is the important role of surgery in the diagnosis and management of fistulizing CD. Examination under anesthesia not only is key to characterizing the anatomy of fistula but also provides opportunity for placement of noncutting Setons which can be an important adjunct intervention to anti-TNF therapy early in the course of the disease that may reduce the risk of perianal abscess secondary to early cutaneous closure. On the other side of the spectrum, surgery also serves an important role as salvage therapy for medically refractory perianal fistula. Fecal diversion and proctectomy may be required in the most severe cases of perianal disease and would ideally be performed by those specializing in colorectal surgery.

A recurring theme of these guidelines is the multidisciplinary nature of managing fistulizing CD. From diagnosis to treatment, expertise in gastrointestinal imaging and surgery are essential components to optimizing care in this patient population. One might argue that caring for CD patients with perianal disease should be done at academic and tertiary IBD centres that have the infrastructure to foster a collaborative model of care. Access to tertiary IBD care will become increasingly important as more novel interventions such as stem cell therapy and hyperbaric oxygen are further developed and formally tested.

The CAG has taken an important step in developing guidelines for the management of fistulizing CD, taking into consideration resources that are available in Canada. These consensus statements will be a valuable resource that will hopefully not only reduce variability in care but also influence provincial health ministries to allocate appropriate resources in order to optimize care.
